# Association between famine exposure in early life with insulin resistance and beta cell dysfunction in adulthood

**DOI:** 10.1038/s41387-020-0121-x

**Published:** 2020-06-08

**Authors:** Yuying Wang, Heng Wan, Chi Chen, Yi Chen, Fangzhen Xia, Bing Han, Qin Li, Ningjian Wang, Yingli Lu

**Affiliations:** Institute and Department of Endocrinology and Metabolism, Shanghai Ninth People’s Hospital, Shanghai JiaoTong University School of Medicine, 200011 Shanghai, China

**Keywords:** Diabetes, Pre-diabetes

## Abstract

**Objectives:**

Famine exposure in early life was associated with type 2 diabetes, non-alcoholic fatty liver disease and metabolic syndrome, etc. But evidence in early famine exposure and insulin resistance and beta cell dysfunction were limited. We aimed to investigate whether the association existed between famine exposure in early life and beta cell dysfunction and insulin resistance in adulthood.

**Methods:**

In all, 7912 non-diabetic participants were included in this study, based on SPECT-China study. Participants with fetal or childhood famine exposure (birth year 1949–1962) were exposure group. Insulin resistance was estimated by the homeostasis model assessment index of insulin resistance (HOMA-IR). Beta cell function, represented by insulin secretion, was estimated by the disposition index. The associations of famine exposure with HOMA-IR and disposition index were assessed via linear regression.

**Results:**

In men, we did not observe a significant association between early life famine exposure and ln(HOMA-IR) in all three models (*P* > 0.05 for all). However, in women, early life famine exposure were found to have significant association with ln(HOMA-IR) after adjustments for urbanization, severity of famine exposure, current smoker, waist circumference, hypertension, and dyslipidemia (unstandardized coefficients 0.055, 95% confidence interval 0.021, 0.088, *P* = 0.001). Early life famine exposure was observed to be negatively associated with ln(disposition index) after adjustments for the above potential confounders, both in men (model 3: unstandardized coefficients −0.042, 95% confidence interval −0.072,−0.012, *P* = 0.006) and women (model 3: unstandardized coefficients −0.033, 95% confidence interval −0.058,−0.009, *P* = 0.008).

**Conclusions:**

In conclusion, exposure to famine in fetal- and childhood- life period is associated with beta cell dysfunction in males and females without diabetes, but early life famine exposure was only associated with insulin resistance in non-diabetic females. These results indicate that malnutrition in early life period may offer a modifiable factor for type 2 diabetes development.

## Introduction

Insulin resistance and beta cell dysfunction are regarded as critical contributors to the progression of several highly prevalent glucose metabolic disorders, including diabetes, metabolic syndrome, and nonalcoholic fatty liver disease (NAFLD)^1–5^. The impaired insulin sensitivity and beta cell function can also influence the risk of lethal complications of the glucose metabolic diseases. It has been proved that lower beta cell function and impaired insulin sensitivity are associated with macrovascular complications^6–8^. Even in individuals without diabetes mellitus, insulin resistance and fasting insulin levels are associated with asymptomatic atherosclerosis and coronary artery disease^9–12^.

The China’s Great Famine from 1959 to 1962, is regarded as the largest and most severe famine during the 20th century^13^. Many reports reported that the famine could have long-term effects for the health condition of early life exposed cohorts. The studies show consistent associations between prenatal famine and adult body size, diabetes, and schizophrenia^14^. These associations could be explained by the Developmental Origins of Health and Disease (DOHaD) hypothesis. DOHaD postulates that adverse events occur during early phases of human development affect the pattern of health and disease throughout life, especially chronic and metabolic diseases^15^. Early exposure to this national calamity was also reported to have close association with NAFLD, metabolic syndrome, and visceral adipose dysfunction in adulthood in our previous studies^16–18^, and was also found to exacerbate the association between hypertension and cardiovascular disease^19^.

Therefore, it provides a possible explanation that famine might cause the metabolic diseases and their fatal complications by impairing insulin sensitivity and beta cell function. However, as beta cell dysfunction and impaired insulin sensitivity may increase the risk of cardiovascular events in non-diabetic individuals, it raises a thought-provoking question whether early life famine exposure is associated with beta cell dysfunction and impaired insulin resistance in non-diabetic population.

A large investigation called the Survey on Prevalence in East China for Metabolic Diseases and Risk Factors (SPECT-China) was conducted in 2014. The aim of this study was to analyze the association between famine exposure in early life and beta cell dysfunction and insulin resistance in adulthood.

## Subjects and methods

### Study population

SPECT-China was a cross-sectional investigation of the prevalence of metabolic diseases and risk factors in East China (ChiCTR-ECS-14005052, www. chictr.org.cn). A stratified cluster sampling method was used to select a sample from the general population. In total, 13,064 subjects were recruited. The exclusion criterion was the following: no blood sample submitted (*n* = 199) and questionnaire data (*n* = 192), younger than 18 years old (*n* = 7), missing fasting plasma glucose (FPG), fasting insulin and HbA1c (*n* = 246), and diagnosed as diabetes (*n* = 1882) for the potential influence of drug use. To achieve age balance between early life famine exposure and non-exposed group, we removed male born after 1972 (*n* = 799) and female born after 1969 (*n* = 1827) because of the diverse age distribution between 2 genders. Finally, 7912 subjects were included in this study. Due to the uncertainty of the exact start and end date of the famine, subjects born in 1962 (*n* = 383) were excluded in sensitivity analysis.

The study protocol was approved by the Ethics Committee of Shanghai Ninth People’s Hospital, Shanghai JiaoTong University School of Medicine. All procedures followed were in accordance with the ethical standards of the responsible committee on human experimentation (institutional and national) and with the Helsinki Declaration. Informed consent was obtained from all participants in the study.

### Clinical, anthropometric, and laboratory measurements

The staff were trained according to a standard protocol that familiarized them with the specific tools and methods used. They used a questionnaire to collect information on participants’ demographic characteristics, medical history, and lifestyle risk factors. Venous blood samples were drawn after an overnight fast of at least 8 h. Blood samples for the plasma glucose test were collected into vacuum tubes with the anticoagulant sodium fluoride and centrifuged within an hour of collection. Blood samples were stored at −20 °C when collected and shipped within 2–4 h of collection by air on dry ice to a central laboratory, which was certified by the College of American Pathologists. Hemoglobin A1c was assessed by HPLC (MQ-2000PT, Medconn, Shanghai, China). Fasting plasma glucose triglycerides, total cholesterol, high (HDL-C) and low-density lipoprotein (LDL-C) were measured by a Beckman Coulter AU 680 analyzer and insulin by chemiluminescence (Abbott i2000 SR).

### Exposure age and area categories

Exposure to famine was based on a proxy, namely the year of birth. Based on a study by van Abeelen et al.^20^, subjects were categorized into two groups according to their age and life stages when exposed to famine from 1 January 1959 to 31 December 1962: fetal (birth year 1959–1962, *n* = 1120) and childhood (birth year 1949–1958, *n* = 2974) famine exposure were combined as early life famine exposure group (birth year 1949–1962, *n* = 4094). For the non-exposed group, in order to choose age balance subjects as the reference, we combined male born before 1949 and 1963–1972 (*n* = 1785), and female born before 1949 and 1963–1969 (*n* = 2033).

The famine severity was determined by the excess death rate for each province and was calculated as the change in mortality rate from the average level in 1956–1958 to the highest value in 1959–1962^21^. Areas of famine were divided into severely (Anhui Province) and moderately (Jiangxi, Jiangsu, Shanghai) affected areas according to the excess death rates during the famine.

### Definition of variables

Insulin resistance was estimated by the homeostasis model assessment index of insulin resistance (HOMA-IR): (fasting insulin [milli international units per liter]) × (FPG [millimoles per liter])/(22.5)^22^. Insulin secretion was estimated by the disposition index, calculated as (20 × fasting insulin [milli international units per liter])/(FPG [millimoles per liter] − 3.5]/HOMA-IR^23^.

Hypertension was identified by a systolic blood pressure (BP) more than or equal to 140 mm Hg, a diastolic BP more than or equal to 90 mm Hg, or a self-reported previous diagnosis of hypertension by a physician.

According to the modified National Cholesterol Education Program-Adult Treatment Panel III, dyslipidemia was defined as total cholesterol more than or equal to 6.22 mmol/L, triglycerides more than or equal to 2.26 mmol/L, LDL-C more than or equal to 4.14 mmol/L or HDL-C < 1.04 mmol/L, or a self-reported previous diagnosis of hyperlipidemia by physicians^24^.

### Statistical analysis

SPSS Statistics, version 21 (IBM Corp) was used to perform the statistical analyses. All analyses were two-sided. *P* < 0.05 was considered statistically significance. Marginal effect was indicated 0.05 < *P* < 0.1. Continuous variables were expressed as the mean ± SD for normal distribution or median (interquartile range) for skewed distribution, and categorical variables were described as a percentage (%). Characteristics of the study sample were compared by the Kruskal–Wallis test and ANOVA for continuous variables with skewed distribution and normal distribution, and Pearson Chi square test for categorical variables.

The associations of life periods when exposed to famine (independent variable) with insulin resistance (HOMA-IR) and beta cell function (disposition index) (dependent variable) were assessed via linear regression. HOMA-IR and disposition index were log transformed to achieve a normal distribution. For the different life stages, the non-exposed group was the reference.

Model 1 was adjusted for urbanization^25^, severity of famine exposure, and current smoker. Model 2 was adjusted for terms for model 1, and waist circumference. Model 3 was adjusted for terms for model 2, hypertension, and dyslipidemia. Data were expressed as unstandardized coefficients (B) (95% confidence interval).

Sensitivity analyses were performed. Because the exact start and end date of the famine was not clear, subjects born in 1962 (*n* = 383) were excluded when performing the regression analyses.

## Results

### Characteristics of the famine-exposed and non-exposed participants

Table 1 shows the results of the variables of the early life famine-exposed and age-balanced non-exposed subjects. In men, compared to the non-exposed subjects, the waist circumference of famine exposed subjects was significantly lower (*P* = 0.049), while the level of HOMA-IR and disposition index, and the prevalence of hypertension and dyslipidemia were comparable between famine exposed and age-balanced non-exposed group. In women, compared to the non-exposed subjects, the famine exposed subjects demonstrated a higher prevalence of dyslipidemia (*P* < 0.001). Moreover, in early life famine exposed women, the level of HOMA-IR was significantly higher while the disposition index were lower compared with non-exposed subjects (*P* ≤ 0.001).

**Table 1:** Characteristics of the early life famine-exposed and non-exposed participants.

	Non-exposed (age balanced)	Early life exposed (1949–1962)	*P* value
Men			
*N*	1785	1583	
Age (when examined), year	59 ± 12.8	59.3 ± 4.2	0.367
Waist circumference, cm	84.74 ± 9.35	84.1 ± 9.03	0.049
Rural/urban residence, %	57/43	51.9/48.1	0.003
Current smoker, %	47	52.6	0.002
Hypertension, %	56.4	55.6	0.644
Dyslipidemia, %	40.7	43.1	0.146
HOMA-IR	1.03 (0.67–1.53)	1.03 (0.69–1.51)	0.895
Disposition index	47.17 (36.81–62.14)	46.48 (35.44–61.59)	0.052
Women			
*N*	2033	2511	
Age (when examined), year	59.1 ± 12.1	59.1 ± 4.1	0.982
Waist circumference, cm	78.99 ± 9.46	79.52 ± 8.79	0.058
Rural/urban residence, %	53.3/46.7	47.6/52.4	0.001
Current smoker, %	3	2.2	0.114
Hypertension, %	50.4	50.4	0.976
Dyslipidemia, %	65.6	55.7	<0.001
HOMA-IR	1.18 (0.82–1.67)	1.27 (0.91–1.8)	<0.001
Disposition index	49.36 (36.57–64.41)	47.17 (35.89–60.52)	0.001

Continuous variables were expressed as the mean ± SD for normal distribution or median (interquartile range) for skewed distribution, and categorical variables were described as a percentage (%). Characteristics of the study sample were compared by the Mann–Whitney U test and independent sample t test for continuous variables with skewed distribution and normal distribution, and Pearson Chi square test for categorical variables.

Fetal (birth year 1959–1962) and childhood (birth year 1949–1958) famine exposure were combined as early life famine exposure group (birth year 1949–1962). Male born before 1949 and 1963–1972, and female born before 1949 and 1963–1969 were age-balanced non-exposed reference.

Homeostasis model assessment index of insulin resistance (HOMA-IR): (fasting insulin [milli international units per liter]) × (FPG [millimoles per liter])/(22.5).

Disposition index, calculated as (20 × fasting insulin [milli international units per liter])/(FPG [millimoles per liter] − 3.5]/HOMA-IR.

### Association of famine exposure with insulin resistance

**Fig. 1:**
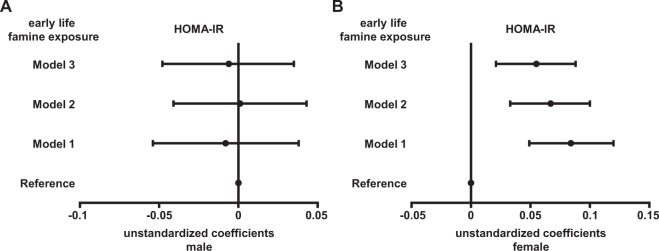
Association between early life famine exposure and HOMA-IR in male and female subjects. Data were unstandardized coefficients (95% confidence interval), which were calculated by linear regression analyses. Fetal (birth year 1959–1962) and childhood (birth year 1949–1958) famine exposure were combined as early life famine exposure group (birth year 1949–1962). Male born before 1949 and 1963–1972, and female born before 1949 and 1963–1969 were age-balanced non-exposed reference. Model 1 was adjusted for urbanization, severity of famine exposure, and current smoker. Model 2 was adjusted for terms for model 1, and waist circumference. Model 3 was adjusted for terms for model 2, hypertension, and dyslipidemia.

Figure 1 demonstrates the association of famine exposure with HOMA-IR in men and women. In men, we did not observe a significant association between early life famine exposure and ln(HOMA-IR) in all three models (*P* > 0.05 for all). However, in women, early life famine exposure were found to have significant association with ln(HOMA-IR) after adjustments for urbanization, severity of famine exposure, current smoker, waist circumference, hypertension, and dyslipidemia (Fig. 1, model 3: unstandardized coefficients 0.055, 95% confidence interval 0.021, 0.088, *P* value = 0.001).

### Association of famine exposure with insulin secretion

**Fig. 2:**
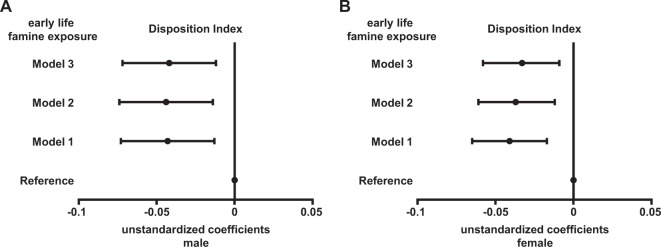
Association between early life famine exposure and Disposition Index in male and female subjects. Data were unstandardized coefficients (95% confidence interval), which were calculated by linear regression analyses. Fetal (birth year 1959–1962) and childhood (birth year 1949–1958) famine exposure were combined as early life famine exposure group (birth year 1949–1962). Male born before 1949 and 1963–1972, and female born before 1949 and 1963–1969 were age-balanced non-exposed reference. Model 1 was adjusted for urbanization, severity of famine exposure, and current smoker. Model 2 was adjusted for terms for model 1, and waist circumference. Model 3 was adjusted for terms for model 2, hypertension, and dyslipidemia.

Figure 2 demonstrates the association of famine exposure with disposition index in men and women. Early life famine exposure was observed to be negatively associated with ln(disposition index) after adjustments for urbanization, severity of famine exposure, current smoker, waist circumference, hypertension, and dyslipidemia (Fig. 2), both in men (model 3: unstandardized coefficients −0.042, 95% confidence interval −0.072,−0.012, *P* value = 0.006) and women (model 3: unstandardized coefficients −0.033, 95% confidence interval −0.058,−0.009, *P* value = 0.008).

### Sensitivity analysis

Due to the uncertainty of the exact start and end date of the famine, subjects born in 1962 (*n* = 383) were excluded to perform the regression analyses. The association did not change between HOMA-IR and the fetal- or childhood-exposed women, as well as disposition index and the fetal- or childhood-exposed subjects (Supplementary Fig. 1).

Age of menopause was also a potential confounder in these associations^26–28^, and we obtained these data in the questionnaire in the latter half of our investigated locations, including 2409 female subjects. We found that after adjustment for urbanization, severity of famine exposure, current smoker, waist circumference, hypertension, dyslipidemia, and age of menopause, early life famine exposure was associated with HOMA-IR (0.078(0.025,0.131), *P* = 0.004), and marginally associated with disposition index (−0.035(−0.075, 0.005), *P* = 0.083).

## Discussion

In this study, we reported a significant association of famine exposure during fetal and childhood with insulin resistance and beta cell dysfunction in non-diabetic population. The association between HOMA-IR and famine exposure in early life was only observed in female, while the association between disposition index and famine exposure in fetal and childhood was observed in both male and female, and were consistent after adjusting for urbanization, severity of famine, current smoking, waist circumference, dyslipidemia, and hypertension. To our knowledge, this is the first study analyzing the association of famine exposure in early life with insulin resistance and beta cell dysfunction in adulthood in non-diabetic individuals. Although previous studies have found that famine exposure was associated with diabetes^18,20,29,30^, our data suggested that famine exposure in early life may be a reason for the pathogenesis of diabetes in later life periods, providing a reliable foundation for the understanding of previous researches.

Evidences have proven that famine exposure during both prenatal and postnatal could contribute to the metabolism disorders in adulthood. Most previous studies focused on prenatal famine exposure and its association with metabolic diseases, and found that for adults prenatally exposed to famine, metabolic dysfunctions in adulthood were at high risk^31–33^. Besides, a meta-analysis performed recently indicated that famine exposure during early life especially fetal-infant exposure might increase the risk of type 2 diabetes in adulthood^34^. For postnatal exposure to famine, we and others suggested that there was a link of exposure to China’s great famine in early life to type 2 diabetes and metabolic diseases^16,18,34–36^. In this study, we found that famine exposure in fetal and childhood showed significantly higher FPG and HbA1c levels, and more prevalent dyslipidemia in both men and women, which was in accordance with previous studies^18,35^, indicating that individuals with postnatal famine exposure in early life were prone to develop metabolic disorders.

Female seems to be more metabolically vulnerable in adulthood after exposure to famine in fetal and childhood. In this study, we found that waist circumference and HOMA-IR were only found higher in female exposed subjects, not in male. Moreover, the association between HOMA-IR and famine exposure were found in female. This gender-specific association has also been found in our previous studies^16–18^. The disorders closely related to insulin resistance, such as NAFLD, metabolic syndrome and visceral fat dysfunction, tended to be found in women, not men^16–18^. Famine early exposure in women could also lead to adverse health conditions^37^. The sex-specific effect reflected an increase of premature deaths among men with famine exposure^13^. The male excess death rate often exceeds that of female’s during famine, particularly in infants, and thus male survivors might be healthier^13^. Moreover, “son preference” in the traditional Chinese value, could make daughters have less food and suffer more, and therefore worsened metabolic outcomes of women in adulthood^13^.

In non-diabetic population, insulin resistance and beta cell dysfunction play a crucial role in the progression to metabolic diseases and cardiovascular diseases. Recently, a real-world setting retrospective observational study found that the incidence of type 2 diabetes is strongly correlated with decline in beta cell function, independent of the therapy used^8^. Progression to type 2 diabetes in people at high risk of diabetes can be markedly reduced with interventions designed to correct underlying pathophysiological disturbances (ie, impaired insulin secretion and resistance)^8^. Furthermore, overproduction of reactive oxygen species, advance glycation end products and further increased low-grade inflammation could be a reason for insulin resistance and hyperglycemia to contribute to an elevated risk of cardiovascular disease^38^. Therefore, insulin resistance and beta cell dysfunction might accelerate the development of metabolic and cardiovascular diseases, so we focused on their association with famine exposure. We found that in non-diabetic individuals, famine exposure in early life was positively and significantly associated with HOMA-IR in female subjects, and was negatively and significantly associated with disposition index in both male and female subjects, indicating that early famine exposure might be a risk factor for insulin resistance and beta cell dysfunction in adulthood in non-diabetic population. Other researchers also found that subjects of prenatal famine exposure, especially during late gestation, was reported to be associated with decreased glucose tolerance in adults^33^. Besides, people prenatally exposed to famine also had higher 2-h glucose concentrations after an oral glucose tolerance test in later life, and seems to be mediated through an insulin secretion defect^39^.

The underline mechanism of insulin resistance and beta cell dysfunction caused by famine exposure remained to be elucidated. There are several possible explanations. First, the DOHaD theory could be a general base of the pathological mechanism^15^. Second, experimental studies have proved that under famine and high-fat refeeding stress, rats were extremely susceptible to develop adverse metabolic events such as hepatic steatosis^40^; beta cell could simultaneously poise to efficiently increase (pro)insulin production upon refeeding after prolonged fasting^41^. In rats with famine stress, the levels of fatty acid synthetase (FAS), sterol regulatory binding-protein 1c (SREBP1c) and glucose-6-phosphate dehydrogenase were elevated^40^. During the famine period, fasted beta cells had marked degranulation, upregulated autophagolysomal and lysosomal organelles, as well as expanded Golgi that correlated with blunted in vivo insulin secretion^41^. Finally, epigenetic changes might participate in this process. Specific epigenetic influences of malnutrition through maternal and early postnatal diet have been observed to be associated with metabolism^42^. Prenatal malnutrition-associated differentially methylated regions were found in *INSR* and *CPT1A*, and their prenatal malnutrition-associated differentially methylated regions^43^.

There are also some limitations that should be mentioned. First, as an observational study, the age between non-famine exposure groups and famine exposure groups were not comparable. The Chinese great famine was a national catastrophe, so we could not identify a group of subjects with comparable age that completely avoid the influence of famine. Secondly, we recorded the urbanization status of the subjects, assuming they did not immigrate to other areas, but bias might exist in this issue. Since permanent residency acquisition always has strict requirements, only 2.68% of the rural population lived in provinces other than their birthplaces^30^. Thirdly, the exact start and end date of the famine was not definite, thus some of the subjects might have been miscategorized into other groups. So we excluded the subjects born in 1962, and the association was still significant (Supplementary Fig. 1). Fourthly, we used HOMA-IR and disposition Index to estimate the condition of insulin resistance and beta cell dysfunction. In large epidemiological study, researchers could not perform examinations such as oral glucose tolerance test or clamp experiments, and HOMA-IR and disposition Index were also widely recognized tools reflecting insulin resistance and beta cell dysfunction^22,23^. Therefore, we applied these two indexes in this study. Finally, menarche is associated with metabolic disorder such as insulin resistance^44^, and early life famine exposure was reported to have effect on menarche^45^. Therefore, menarche could be confounders for the association of early life famine exposure with insulin resistance and beta cell dysfunction. However, age of menarche was not recorded in this study. Future investigation should be performed to further analyze this issue.

In conclusion, we reported that famine exposure in early life was positively and significantly associated with HOMA-IR in female subjects, and was negatively and significantly associated with disposition index in both male and female subjects. These associations may partly explain both the sex-specific association between early famine and adverse metabolic conditions in women, such as NAFLD, metabolic syndrome and visceral fat dysfunction in women, and the association between early famine and type 2 diabetes in men. The association between early famine exposure with insulin resistance and beta cell function should be confirmed in further studies, and the underlined mechanism still calls for exploration.

## Supplementary information

Sensitivity analysis
