# Supplementary material for: Association between famine exposure in early life with insulin resistance and beta cell dysfunction in adulthood
Source: Nutr Diabetes. 2020 Jun 8;10:18. doi: 10.1038/s41387-020-0121-x (PMC7280514; doi:10.1038/s41387-020-0121-x)
Supplement: Supplementary file 1 — Sensitivity analysis [file 41387_2020_121_MOESM1_ESM.docx]

**Supplementary Figure 1 Association of famine exposure with the HOMA-IR and Disposition Index in subjects except for those born in 1962.**


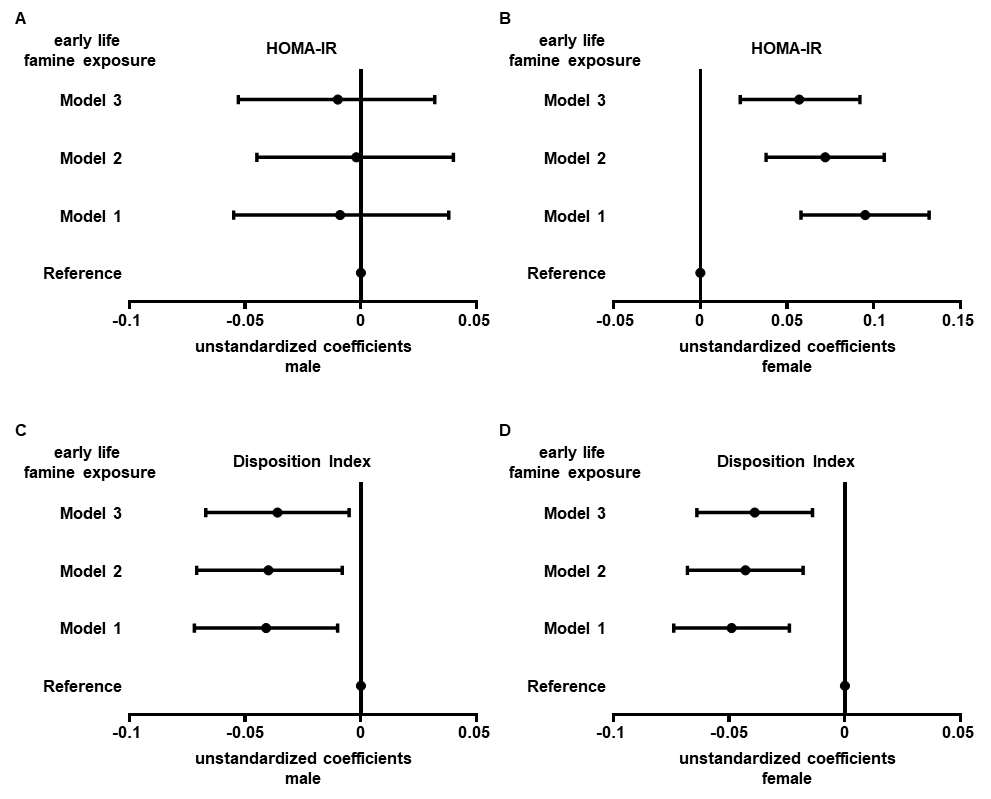


Data were unstandardized coefficients (95% confidence interval), which were calculated by linear regression analyses.

Fetal (birth year 1960-1961), childhood (birth year 1949-1958), and fetal/childhood (birth year 1959) famine exposure were combined as early life famine exposure group (birth year 1949-1961). Male born before 1949 and 1963-1972, and female born before and 1963-1969 were age-balanced non-exposed reference.

Model 1 was adjusted for urbanization, severity of famine exposure, and current smoker. Model 2 was adjusted for terms for model 1, and waist circumference. Model 3 was adjusted for terms for model 2, hypertension, and dyslipidemia.
